# Progress in Surgery

**Published:** 1897-02-20

**Authors:** 


### PROGRESS IN SURGERY.

### ORTHOPÆDIC SURGERY.

**The Scope of Orthopædic Surgery.**—Dr. Royal Whitman[1] defines orthopædic surgery thus: Orthopædic surgery is that division of surgery which treats of the locomotive apparatus and of the prevention and treatment of deformities of the framework of the body. He says that orthopædic surgery inherits an ancient name, and the traditions of a still more ancient practice, but it is no longer bound to those traditions. It no longer separates deformities from its causes, nor divides it into classes to fit it to a peculiar therapeutic method. But it now maintains, in common with other specialities, that a knowledge of cause and effect, modified it may be by practical experience and the individual needs of the patient, is the only safe and enduring standard upon which treatment may be conducted. He disavows the purely mechanical method of treating deformities, and advocates most strongly the proper surgical treatment, claiming also that orthopædic surgery be preventive as well as curative. The same author,[2] discussing a theory of the ultimate etiology of deformity, says, if, from any cause, voluntary control of the attitudes or motions of a limb is weakened, and the influence of the lower or reflex centres is relatively or absolutely increased, the affected member will tend to assume an habitual attitude, resembling in degree that proper to a lower or quadrupedal type of locomotion. The reflex muscular spasm that is present in chronic joint disease is a symptom of local irritation and weakness, and flexion deformity is an evidence of unbalanced nervous energy and loss of cerebral control. This flexion, at first a symptom, becomes a fixed and permanent deformity by the degenerative changes in the contracted muscle, and by accommodative changes in and about the diseased joint.

**Deformity of the External Ear.**—J. F. Binnie,[3] after describing the development of the external ear as a series of buds arising from the first, and especially the second, branchial arch, remarks that a more or less complete failure of union of the auricular buds may result in fistula or dermoids of the pinna, but more especially a condition where the external ear is composed of a series of tags of skin which may or may not contain cartilage, the external auditory meatus is usually absent. He gives a photograph of this deformity, microtia; and adds that in cases of microtia absolute deafness is uncommon; and, conversely, it is true that microtia is uncommon in cases of deaf-mutism. This author also gives a photograph of another and rarer deformity, in which the auricle hung downwards and was located near the angle of the jaw.

**Shoulder Braces.**—C. B. Keetley[4] describes a new form of shoulder-brace, which has met with considerable success in his hands. He says an essential requirement for good shoulder-braces is a firm basis, such as a plaster of Paris or a poroplastic jacket fitted to the trunk as low as the hips. Another is that the braces should not pull or press downwards at all, because that action, as Sayre points out, tends, by bringing the two ends of the spinal column together, to increase spinal curvature. He recommends anterior crutches or braces only. The ordinary axillary crutch, inferior in position, is only endured by the patient when it does not act; when it does, it is well known to paralyse the brachial plexus, and obstruct the circulation in the arm. A third requirement of the anterior crutch or shoulder brace is that its support should be elastic; and a fourth, that the degree of pressure should be adjustable by the patient according to what experience shows can be endured with comfort. Shoulder braces, as described by Mr. Keetley, are anterior in position, and consist of two vertical arms, so arranged that they adjust themselves along the inner border of the deltoids.

**A Special Affection of the Vertebral Column of Traumatic Origin.**—Dr. Kümmell[5] described in 1891 five cases of a peculiar affection of the vertebral column, which consists in the production of a hump without any connection with Potts' disease. The deviation of the vertebral column, which is always consecutive to injury, is associated with various nervous disturbances, but does not give rise to a wandering abscess. During the last year Dr. Kümmell met with another case. Recently, Henle has reported four more cases of the same kind. The cause is usually a severe contusion of the bodies of the vertebræ, which may be determined directly or indirectly; for example, the result of a heavy weight striking the shoulders or posterior cervical region. There are three stages in the affection. In the first stage there is more or less severe rachialgia, involving a variable number of vertebræ, and resulting in functional impotence of the limbs. These symptoms having persisted for some time, usually from two to ten days, gradually disappear. Their disappearance is frequently so complete that the patient may even be enabled to resume work, this constituting the second stage, that of incomplete cure. The third stage is ushered in after a period varying between a few weeks and several months by a recurrence of the pain in the spinal region, soon followed by intercostal neuralgia and motor or sensory disturbance in the limbs. Lastly, deviation of the vertebral column supervenes in the form of a hump, which is tender to pressure, and involving as a rule the dorsal vertebræ. At other times there are scoliosis and flattening of the physiological kyphosis. In one case under Henle's observation there was scoliosis of the lower cervical and upper dorsal vertebræ. If the patient is suspended the deviation of the spinal column disappears, but reappears so soon as suspension ceases, a fact which proves that the tissue of the injured vertebræ has become softer than normal. Taking into account the history of the patient, the comparatively early appearance of the deviation of the vertebral column after the injury, and the absence of tuberculous lesions in other organs and of a burrowing abscess, it is an easy matter to distinguish the affection described by Kümmell and Henle from Potts' disease. Mikulicz and Henle, however, are of opinion that the osseous lesion is the result of vaso-motor and trophic disturbances due to pachymeningitis following the injury to the vertebral column.

Schapps,[6] after describing his apparatus for the treatment of Potts' Disease, goes on to state that in spinal cases the weight of the parts above is by no means all that the diseased bone has to bear. There is also that of the parts which, though situated below, are suspended from above. The viscera of the abdomen make downward traction on the spine at a much higher level than their own. The upper extremities are, by means of muscles extending upwards from the clavicles and scapulæ, suspended from the head and the cervical vertebræ. So, too, are the thorax and the abdominal muscles. The diseased spine is subject also to muscular tension exerted between the parts above and those below, by reason of the forward projection of the face, chest, and pelvis; much of this force is applied in a deforming manner and at a great leverage.

[1] New York Medical Journal, June 20th, 1896. [2] Annals of Surgery, Aug., 1896. [3] Ibid. [4] Brit. Med. Jour., May 30, 1896. [5] The Med. Week, April 10, 1896. [6] New York Med. Jour., Aug. 1, 1896.

